# Establishment of a human nasal epithelium model of histamine-induced inflammation to assess the activity of fexofenadine as an inverse agonist and its link to clinical benefit

**DOI:** 10.3389/fphar.2024.1393702

**Published:** 2024-06-12

**Authors:** Anne Barbot, Michele Lheritier-Barrand, Margarita Murrieta-Aguttes, Maud Leonetti, Jimmy Vernaz, Song Huang, Samuel Constant, Bernadett Boda

**Affiliations:** ^1^ Sanofi, CHC Scientific Innovation, Neuilly, France; ^2^ Sanofi R&D, Vitry-sur-Seine, France; ^3^ Epithelix, Plan-les-Ouates, Switzerland

**Keywords:** inflammation, allergy, nasal epithelium, antihistamine, fexofenadine, biomarkers, inverse agonist

## Abstract

**Background:**

Fexofenadine (FEX) is an antihistamine that acts as an inverse agonist against histamine (HIS) receptor 1 (H1R), which mediates the allergic reaction. Inverse agonists may be more potent than neutral antagonists, as they bind the same receptor as the agonist (HIS) but stabilize the inactive form and induce an opposite pharmacological response, suppressing the basal activity of H1R and preventing HIS from binding. This study aims to establish and validate a model of HIS-induced inflammation based on fully reconstituted human nasal epithelial tissue to assess the activity of FEX as an inverse agonist in this model and explore its link to clinical benefit.

**Methods:**

The model was developed using nasal MucilAir™ (Epithelix) *in vitro* epithelium challenged by HIS. Two conditions were assessed in a side-by-side comparison: tissue was exposed to HIS + FEX with or without FEX pre-treatment (one-hour prior to HIS challenge). Tissue functionality, cytotoxicity, H1R gene expression, and inflammatory cytokines were assessed.

**Results:**

HIS at 100 µM induced significant 3.1-fold and 2.2-fold increases for inflammatory biomarkers interleukin (IL)-8 and IL-6, respectively (*p* < 0.0001), as well as rapid upregulation of H1R mRNA. Inflammatory biomarkers were inhibited by FEX and H1R expression was significantly reduced (*p* < 0.0001). FEX alone decreased H1R expression at all doses tested. With one-hour FEX pre-treatment, there was significantly higher downregulation of IL-8 (*p* < 0.05) and further downregulation of H1R expression and IL-6 *versus* without FEX pre-treatment; the effects of FEX were improved from 22% to 40%.

**Conclusion:**

A model of HIS-induced airway inflammation was established based on IL-8, IL-6 and H1R gene expression and was validated with FEX. FEX works as an inverse agonist, with a higher effect when used before+during *versus* only during the HIS challenge. Taking FEX before+during allergen exposure, or when symptoms first occur, may reduce basal activity and H1R gene expression, providing stronger protection against the worsening of symptoms upon allergen exposure.

## Graphical Abstract

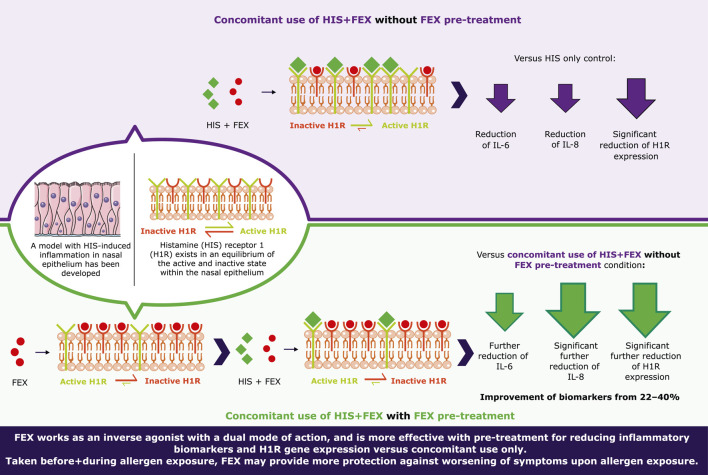

## 1 Introduction

Nasal mucosal inflammation and the associated allergic rhinitis (AR) symptoms are induced by allergic reactions which are largely mediated by histamine (HIS), a major chemical mediator, and its interaction with histamine receptor 1 (H1R) (Powell et al., 2007; Mizuguchi et al., 2012); the activation of H1R by HIS results in the symptoms of AR (Mizuguchi et al., 2012). HIS has been described to upregulate the gene expression of H1R in cells, increasing the strength of H1R signaling and, subsequently, the severity of symptoms (Mizuguchi et al., 2012; Mizuguchi et al., 2021). For example, nasal symptoms of an allergic reaction have previously been associated with an increase of the H1R mRNA in the nasal mucosa of patients (Kitamura et al., 2015), and it has been reported that the severity of AR symptoms is correlated with higher levels of H1R gene expression (Panliang, 1988; Mizuguchi et al., 2012; Mizuguchi et al., 2021). Furthermore, it has been reported that the level of H1R mRNA expression is increased in patients with AR (Mizuguchi et al., 2012). Additionally, the amount of HIS present on the surface of the inferior turbinate of the nose is correlated with the degree of the reaction to provocation, and, interestingly, the lowest amount of HIS required to induce sneezing is lower in allergic patients *versus* healthy controls (Ohtsuka and Okuda, 1981; Iriyoshi et al., 1996). Both HIS level and H1R expression are important factors that mediate the allergic reaction in nasal mucosa (Ohtsuka and Okuda, 1981; Iriyoshi et al., 1996; Kitamura et al., 2015) and, subsequently, AR symptoms. The allergic response is also mediated by cytokines (extracellular signaling proteins) such as chemokines like RANTES (regulated upon activation, normal T-cell expressed and presumably secreted) or interleukins (ILs). Some inflammatory cytokines, including interleukins (such as IL-6 and IL-8), have been consistently reported to be upregulated or increased throughout the allergic inflammatory response and could potentially be used as biomarkers for the allergic response (Ferreira, 2003).

**FIGURE 1:**
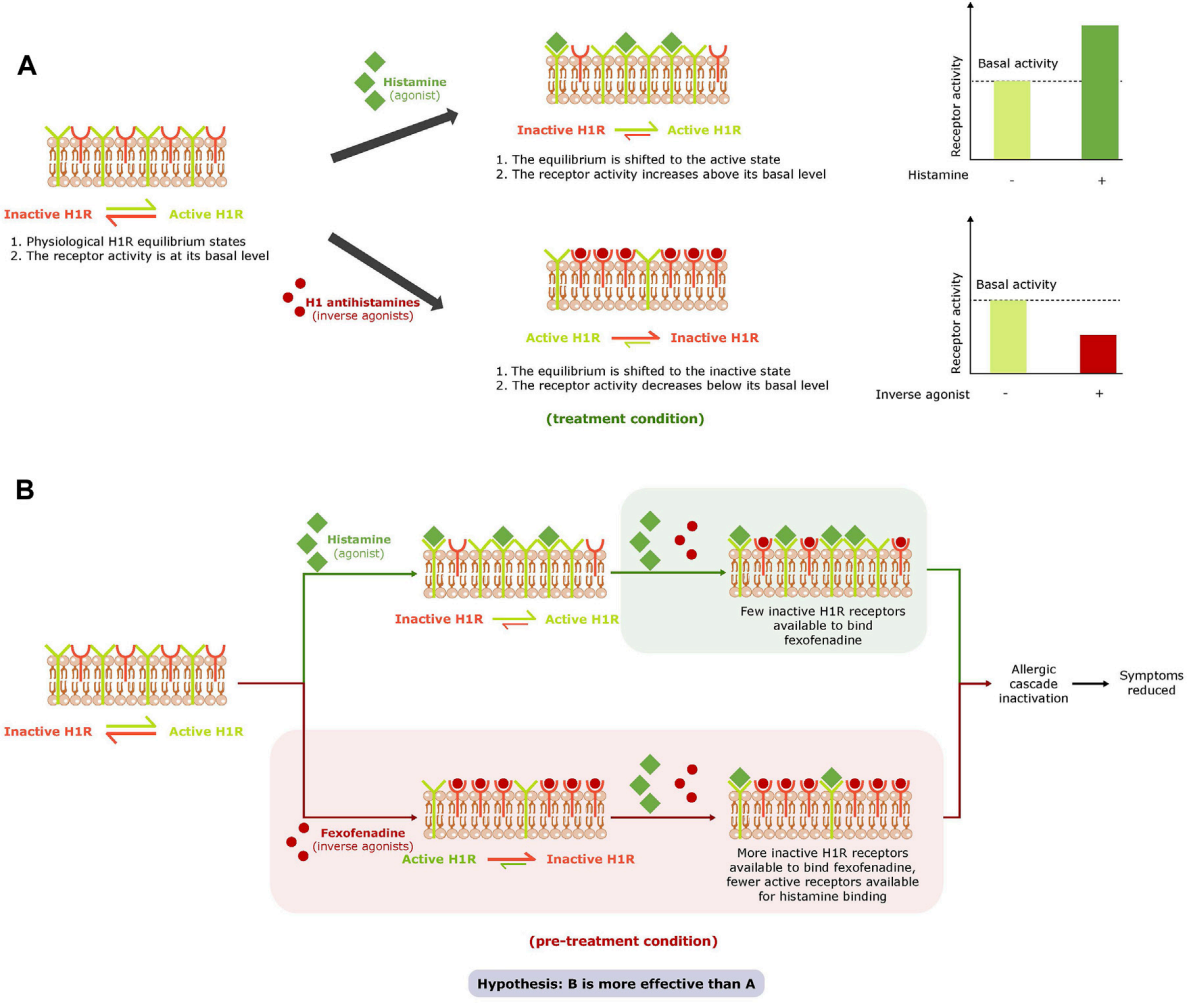
Effect of agonists and inverse agonists on the H1R equilibrium and basal activity **(A)** and study hypothesis **(B)**. abbreviation: H1R, histamine receptor 1.

H1R is described as having some constitutive receptor activity even in the absence of HIS and exists in an equilibrium of an active and inactive state (Leurs et al., 2002; Mizuguchi et al., 2012). When HIS is present at H1R it acts as an agonist and combines with and stabilizes H1R in its active state to shift the equilibrium of H1R towards the active state and activate the HIS-signaling cascade (Leurs et al., 2002). H1-antihistamines prevent the activation of H1R by HIS by binding to H1R through a neutral antagonist or inverse agonist mechanism of action. Some H1-antihistamines act as inverse agonists and reduce constitutive receptor activity by binding with and stabilizing the inactive form of H1R in the absence of HIS, thereby shifting the equilibrium of H1R toward the inactive state (Leurs et al., 2002) (Figure 1A). As such, they may have a higher potency than neutral antagonists as they exhibit a dual mode of action: downregulation of constitutive H1R activity as well as prevention of H1R activation by HIS (Leurs et al., 2002; Mizuguchi et al., 2012; Mizuguchi et al., 2020). Fexofenadine (FEX), a non-sedating antihistamine, is used to relieve AR and urticaria symptoms (Leurs et al., 2002; Meltzer et al., 2021; Ansotegui et al., 2022). FEX is described in the literature as an inverse agonist (Leurs et al., 2002; Meltzer et al., 2021), and based on this mode of action and the fact that H1R is described as having basal activity, it may be assumed that FEX is more effective when it is present prior to H1R activation by HIS than when it is not (Figure 1B). The protective effect of FEX as an inverse agonist is yet to be shown, although the prophylactic use of antihistamines is recommended in current guidelines when patients know they will be exposed to allergens that will aggravate their AR (Bousquet et al., 2008; ACAAI, 2017; American Academy of Allergy Asthma & Immunology AAAACI, 2021; MedlinePlus, 2022).

Studying the effect of compounds on airway cells *in vitro* can be done using reconstituted human nasal epithelial tissue. Such models are used for many acute, long-term, and chronic *in vitro* studies (Constant et al., 2013). However, to our knowledge, there are currently no human nasal tissue-based models of HIS-induced inflammation with measurable biomarkers that can be used to investigate the anti-inflammatory activity of antihistamines and the benefit of inverse agonists in pre-treatment. As such, an *in vitro* model that replicates the main function of airway epithelial cells and allergy-related inflammation is of interest.

The first part of this study was to develop a novel, specific *in vitro* model of HIS-induced inflammation based on fully reconstituted human nasal epithelium tissue and to validate this model by selecting the relevant biomarkers for HIS-induced inflammation and evaluating the effect of antihistamines —FEX and bilastine (BIL) — on these biomarkers. The main aim of this study was, for the first time in this validated model, to test the hypothesis presented in Figure 1B. The study aims to examine the benefit of FEX as an inverse agonist able to exhibit dual actions: inhibition of basal activity of H1R and its activation by HIS. The study also aims to examine the benefit of higher activity when FEX is exposed to the nasal tissue ahead of and during an HIS challenge *versus* during HIS challenge only by shifting the equilibrium of H1R to the inactive state prior to activation by HIS.

## 2 Materials and methods

### 2.1 Chemicals

The HIS (Sigma) stock solution of 1 M was prepared in water, and 100-fold serial dilutions were performed in MucilAir™ culture medium (EP04MM, Epithelix) to obtain test concentrations.

Pure fexofenadine hydrochloride powder was provide by Sanofi. A 1 M stock solution was prepared in dimethylsulfoxide (DMSO), and 100-fold serial dilutions were performed in a MucilAir™ culture medium to obtain test concentrations.

BIL (Selleckchem) 50 mM stock solution was prepared in DMSO, and 10-, 50-, and 100-fold serial dilutions were performed in MucilAir™ culture medium to obtain test concentrations.

All solutions were stored at −20°C.

### 2.2 Reconstituted human nasal epithelium in air-liquid interface (ALI) culture (MucilAir™)

**FIGURE 2:**
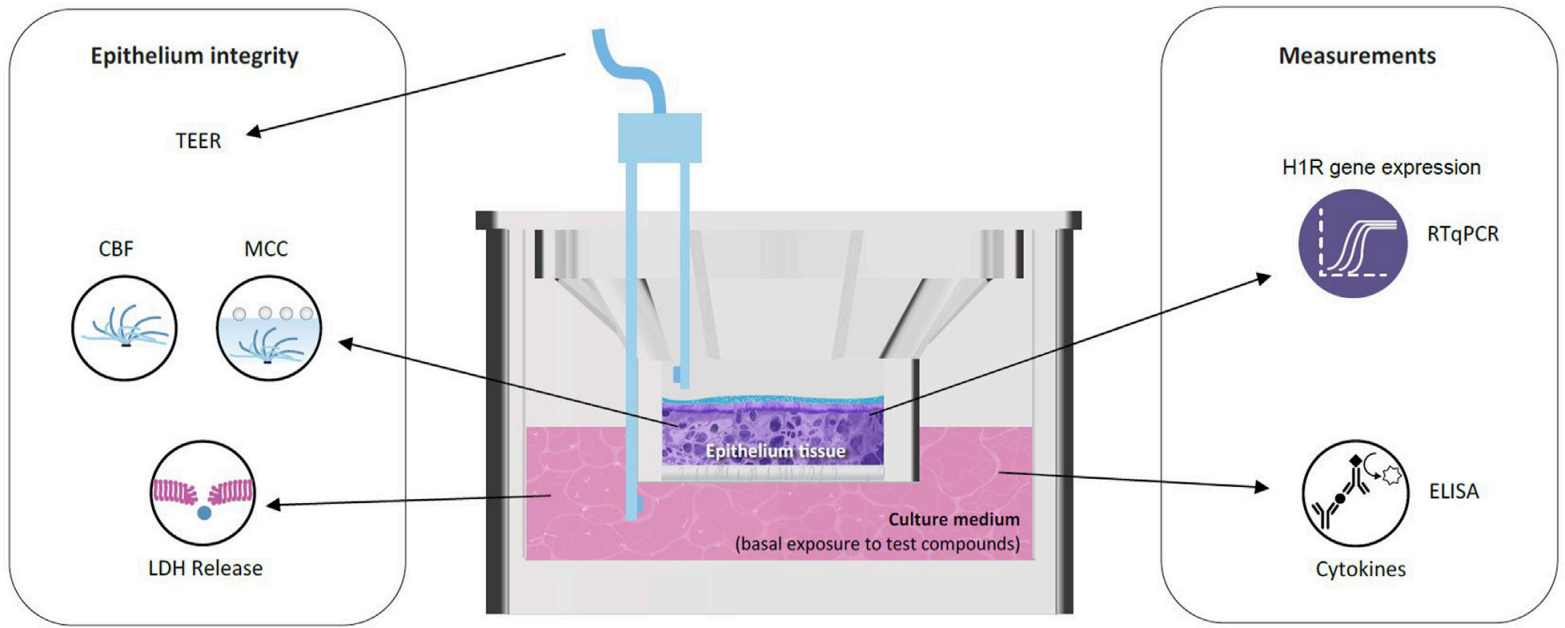
Reconstituted human nasal epithelium model (MucilAir™) to study the effect of HIS and FEX. CBF, cilia beating frequency; ELISA, enzyme-linked immunosorbent assay; LDH, lactate dehydrogenase; MCC, mucociliary clearance; RT-qPCR, quantitative reverse transcription–polymerase chain reaction; TEER, trans-epithelial electrical resistance.

The *in vitro* nasal epithelium (“MucilAir,” http://www.epithelix.com/products/mucilair) was derived from primary human nasal cells obtained from 14 adult patients undergoing surgical nasal polypectomy. Procedures were compliant with good clinical practice guidelines. Primary human nasal cells were expanded once and seeded on Transwell^®^ inserts (24-well format) in the MucilAir™ culture medium. Once confluent, cultures were switched to an ALI culture for at least 28 days to obtain completely differentiated epithelia (MucilAir™-pool). The average culture time after the ALI step was 43 days. Figure 2 and Table 1 display the experimental setup and summarize the collection of the following endpoints. Eight series were conducted.

**TABLE 1:** Study design and endpoint measurement.

Time (hours)			−1	0	1	6	24	48	72	96
CONDITION	Without FEX pre-treatment	HIS	-	+	-	-	+	+	+	-
		FEX	-	+	-	-	+	+	+	-
	With FEX pre-treatment	HIS	-	+	-	-	+	+	+	-
		FEX	+	+	-	-	+	+	+	-
MEASUREMENT	LDH		-	-	-	-	+	+	+	+
	TEER		-	-	-	-	+	+	+	+
	MCC, CBF		-	-	-	-	-	-	-	+
	Cytokine(s)		-	-	-	-	+	+	+	+
	H1R gene expression		-	-	+	+	+	-	-	-

CBF, cilia beating frequency; LDH, lactate dehydrogenase; MCC, mucociliary clearance; TEER, trans-epithelial electrical resistance.

Cytokines measured were IL-6, IL-8, IL-25, IL-33, and thymic stromal lymphopoietin. Control experiments were taken as baseline measures. Gray shading indicates that the first three repetitions of the nine-repetition series lasted 4 days; the remaining repetitions lasted 2 days.

### 2.3 Histology

Two MucilAir™-pools were processed for histology using four central transversal paraffin sections of 4 µm. Hematoxylin and eosin staining (H&E) with Alcian blue (AB), which stains goblet cells, were performed for the qualitative aspect of the tissues. A primary antibody, Mucin 5AC, and a biotinylated secondary antibody, Dako, were used.

### 2.4 Western blot

Four MucilAir™ cultures were lysed together on ice using a 150 µL solution containing 150 mM NaCl, 50 mM Tris-HCl pH = 7.4, 2 mM ethylenediaminetetraacetic acid (EDTA), 1 mM dithiothreitol (DTT), 1% Triton X-100, 10% glycerol, and complete protease inhibitor (Roche, Merck). Total protein lysates (30 μg) were loaded on Mini-Protean Tris-Glycine eXtended gel (BioRad), run in Tris/Glycine/SDS buffer (BioRad), and transferred to nitrocellulose membranes. H1R immunoblotting was performed using a 1:500 dilution of the rabbit polyclonal anti-H1R antibody (LS-C331459, LSBio). Data were normalized by a 1:20,000 dilution of glyceraldehyde 3-phosphate dehydrogenase (GAPDH) (G9545, Sigma) immunoblotting.

### 2.5 Trans-epithelial electrical resistance (TEER) measurement

Saline solution was added to the apical air surface of MucilAir™ cultures for TEER measurement using an epithelial voltohmmeter (EVOMX) (World Precision Instruments, UK), and the apical fluid was removed immediately afterward. Resistance values (Ω) were converted to TEER (Ω.cm^2^) using the following formula, where 100 Ω is the resistance of the membrane and 0.33 cm^2^ is the total surface of the epithelium: TEER (Ω.cm^2^) = (resistance value (Ω) − 100(Ω)) × 0.33 (cm^2^).

### 2.6 Cilia beating measurement

Cilia beating frequency (CBF) was measured via a high-speed camera (125 frames per second) connected to an optical microscope, capturing 256 images at room temperature. Frequency was calculated using Cilia-X software (https://www.epithelix.com/services/cilia-x-cbf-analysis).

### 2.7 Mucociliary clearance (MCC) measurement

Polystyrene microbeads (Sigma) were added to the apical surface of MucilAir™, and their movements were recorded with an optical microscope. Videos (three per culture) were captured at two frames per second for 30 images at room temperature. The average bead velocity (μm/sec) was calculated with the Image-Pro Plus 6.0 software.

### 2.8 Cytotoxicity assay

The basolateral medium was collected and measured using a Cytotoxicity Lactate Dehydrogenase (LDH) Assay Kit, following the manufacturer’s instructions (WST Dojindo, CK12-20). To determine the percentage of cytotoxicity, the following equation was used where A = absorbance values: cytotoxicity (%) = (A (exp value) − A (low control)/A (high control) − A (low control)) × 100. Positive control of cytotoxicity was obtained by apical treatment with 10% Triton X-100. Triton X-100 causes a massive LDH release and corresponds to 100% cytotoxicity.

### 2.9 Effect of HIS and antihistamines on the reconstituted human nasal epithelium (MucilAir™-pool)

Basolateral challenge of HIS (from 1 to 100 µM), with or without FEX or BIL (0.01 µM, 1 µM, or 100 µM), on reconstituted human nasal epithelium in ALI (MucilAir™-pool) was tested. FEX alone and DMSO served as controls. Additionally, HIS alone and concomitant use of HIS + FEX with or without pre-treatment with FEX were assessed in a side-by-side comparison, and concomitant use of HIS + BIL was assessed.

For the standard condition, HIS + FEX or HIS + BIL was added to the culture medium at 0 and 24 h without FEX pre-treatment (referred to as “without FEX pre-treatment” in the results). To assess the effect of FEX pre-treatment*,* FEX was added to the culture medium 1 week, 24 h, or 1 h before the standard condition (referred to as “with FEX pre-treatment” in the results; Table 1). Media were renewed daily for 4 days.

### 2.10 Enzyme-linked immunosorbent assay (ELISA)

The releases of interleukin (IL)-6, IL-8, IL-25, IL-33, and thymic stromal lymphopoietin (TSLP) were measured using ELISA assays according to the manufacturer’s instructions (BD Biosciences, R&D systems). The basolateral culture medium of MucilAir™ was collected for measurement at 24 h, 48 h, 72 h, and 96 h.

### 2.11 RNA extraction and reverse transcription polymerase chain reaction (RT-qPCR) measurement

H1R expression was quantified by TaqMan RT-qPCR using GAPDH as the housekeeping gene. RNA extraction was performed using an RNeasy Mini kit, supplemented by DNase treatment (Qiagen). Transcripts were amplified by QuantiTect Probe RT-PCR kits (Qiagen) and TaqMan Gene Expression Assays (Thermo Fisher Scientific). PCR reactions were performed for each gene in 96-well plates using a qTower Detection System (Analytik Jena). After the automatic threshold of the fluorescence signal, the threshold cycle number (Ct) was used for quantification. The relative amount of transcripts was obtained using the 2^−ΔΔCt^ method, and data were expressed as fold change *versus* the control condition. MucilAir™ was collected for measurement at different time points: 1 h, 3 h, 6 h, or 24 h after concomitant use of HIS + FEX or FEX alone.

### 2.12 Statistical analysis

Data were expressed as mean ± standard error, and statistical analysis was performed by one-way ANOVA with Dunnett’s or Sidak’s multiple comparison post-tests using Prism 6 GraphPad software or by Student’s t-test.

## 3 Results

### 3.1 Characterization of the reconstituted human nasal epithelium (nasal MucilAir™-pool)

**FIGURE 3:**
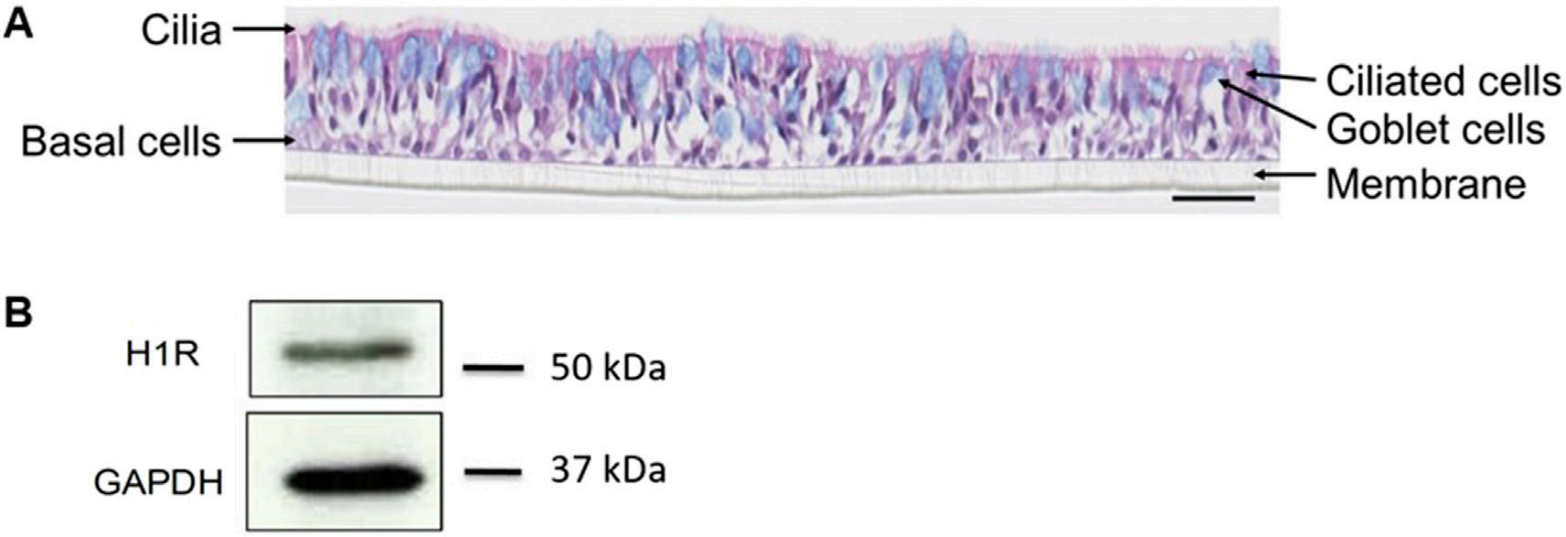
MucilAir™ characterization by histological staining and Western blot. Differentiation of the tissue was observed by **(A)** hematoxylin eosin and alcian blue staining, and **(B)** expression of H1R was shown by Western blot. GAPDH, glyceraldehyde 3-phosphate dehydrogenase; H1R, histamine receptor 1. Scale bar is 50 µm.

**FIGURE 4:**
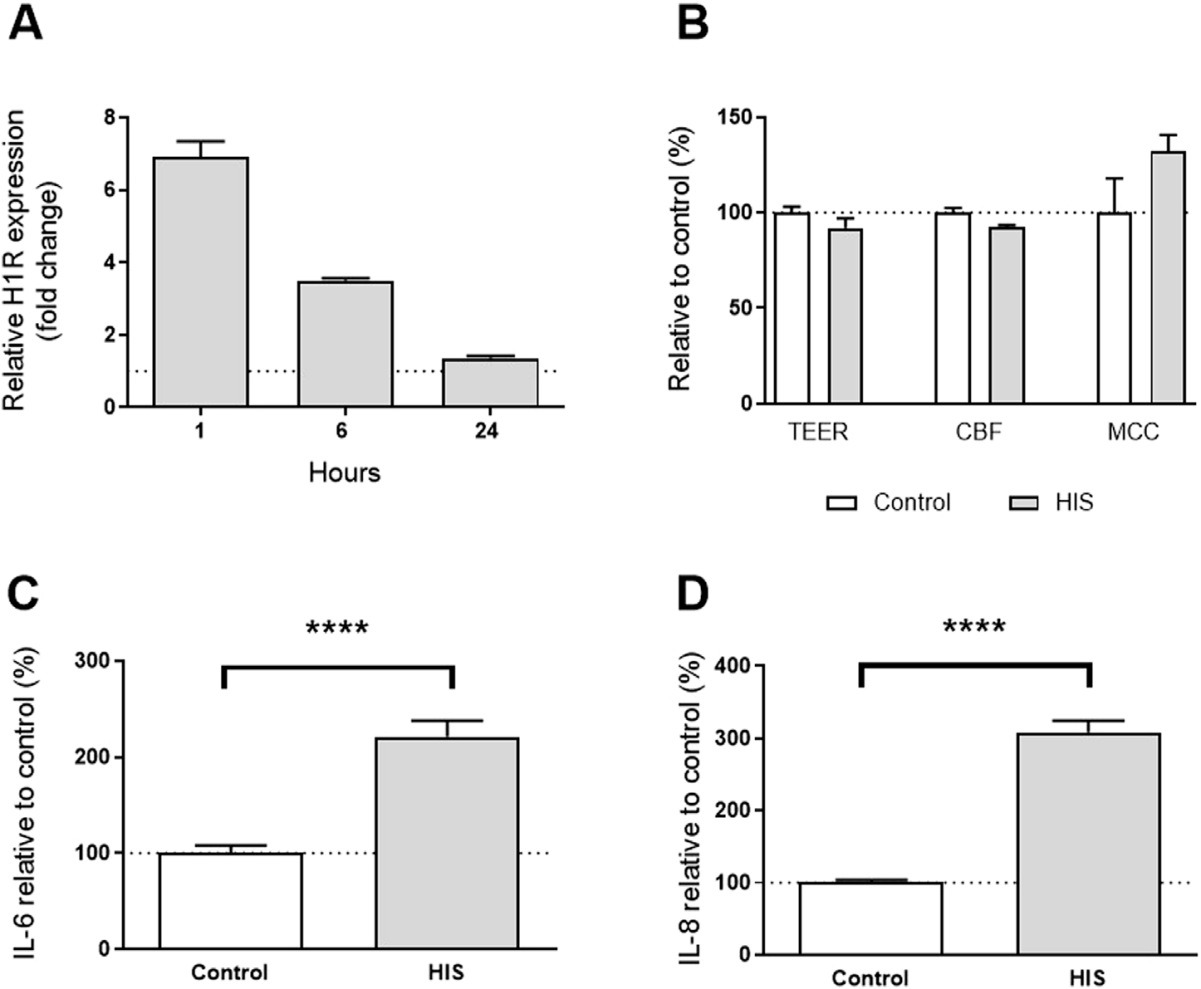
Effect of 100 µM HIS on the nasal MucilAir™-pool. **(A)** H1R gene expression level in MucilAir™-pool tissue after a single challenge of 100 µM HIS at 1 h, 6 h, and 24 h by RT-PCR (n = 3 cultures, mean + SEM); **(B)** tissue integrity (TEER, day 2), cilia beating frequency (CBF, day 4), and mucociliary clearance (MCC, day 4). The control was untreated cultures (n = 4, mean + SEM, Student’s t-tests, *p* > 0.05); **(C)** IL-6 deregulation after 2 days of basal challenge with HIS 100 µM (eight independent experiments, n = 51 cultures, unpaired *t*-test); **(D)** IL-8 deregulation after 2 days of basal challenge with HIS 100 µM (eight independent experiments, n = 51 cultures, unpaired *t*-test). ****p < 0.0001.

The pseudostratified nasal tissue culture contained ciliated cells (active cilia visible on the top), mucin-producing goblet cells (blue cells as observed by Alcian blue staining), and basal cells (Figure 3A) similar to those existing *in vivo*. The expression of H1R in the reconstituted human nasal epithelium was confirmed at protein level by Western blot (Figure 3B) and at RNA level by RT-PCR (Figure 4A). The reconstituted human nasal epithelium (MucilAir™-pool) replicated the main function of airway epithelial cells (Huang et al., 2011); there was full functionality of the epithelial tissue as shown by TEER, CBF, and MCC (Figure 4B), as well as mucus production (data not shown). The MucilAir™ cultures showed a low daily basal LDH release (<5%) (Supplementary Figure S1), showing a physiological steady-state cell turnover in the model.

These results demonstrate that the human nasal epithelium was successfully reconstituted, fully functional and expressing the receptor of interest, H1R.

### 3.2 HIS responses in the *in vitro* human nasal epithelium model (nasal MucilAir™-pool)

The maximum tolerated dose of HIS challenge for which there was no cytotoxicity was 100 µM (data not shown); no change in tissue integrity after 48 h, cilia beating or mucociliary clearance after 96 h of HIS challenge was observed (Figure 4B).

This concentration of HIS induced significant 3.1-fold and 2.2-fold increases of inflammatory cytokines IL-8 and IL-6, respectively (*p* < 0.0001; Figures 4C, D). Additionally, there was a rapid upregulation of the H1R mRNA with a 6.9-fold increase at 1 h and a 3.5-fold increase at 6 h, which returned to the basal level at 24 h (Figure 4A). These data demonstrate that basolateral challenge of HIS at 100 µM induced a pro-inflammatory cytokine release in nasal *in vitro* epithelium correlated with a rapid upregulation of H1R gene expression. No constitutive secretion of IL-25, IL-33, or TSLP was detected in the epithelium, and no change was observed when 100 µM HIS was used (data not shown). IL-6, IL-8, and H1R gene expression were selected as the most relevant biomarkers for this model.

### 3.3 Effect of FEX on *in vitro* human nasal epithelium model of HIS-induced inflammation in different conditions

Concentrations of FEX from 0.01 to 100 µM were tested for concomitant use with 100 µM HIS without FEX pre-treatment. FEX at 1 µM was well tolerated; there was no change in tissue integrity and no cytotoxicity (Supplementary Figure S1). This concentration was then selected as the test concentration for both FEX and BIL to validate the model (without antihistamine pre-treatment).

**FIGURE 5:**
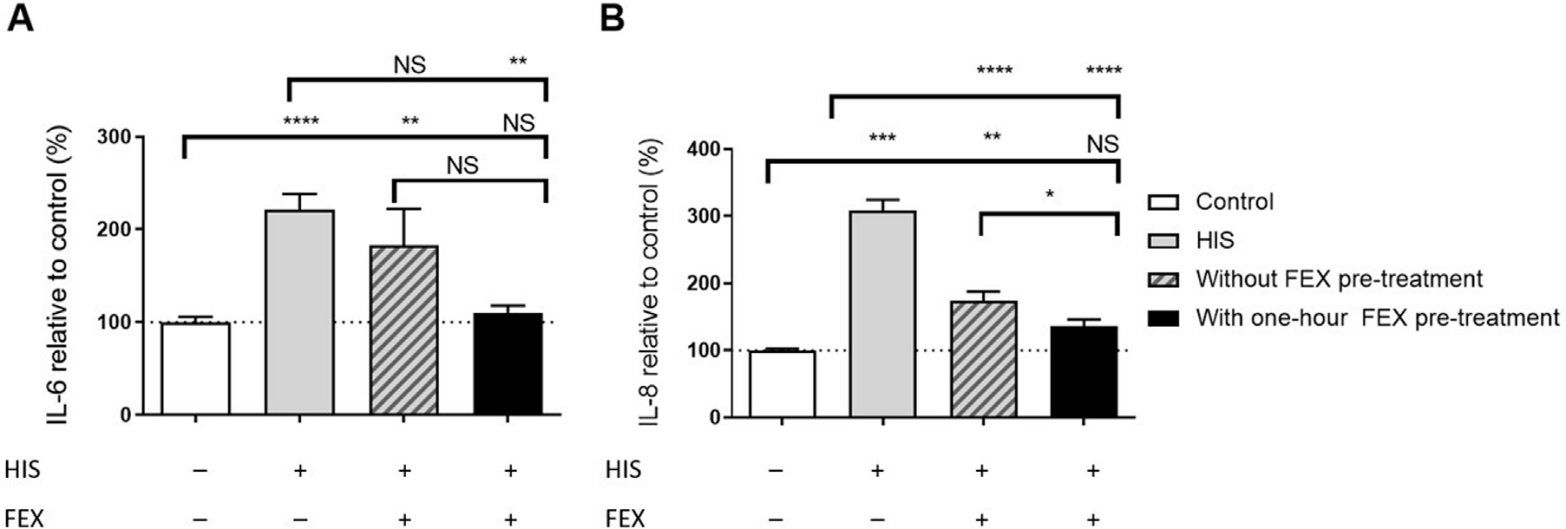
Side-by-side comparison of the anti-inflammatory effect of FEX on IL-6 **(A)** and IL-8 **(B)** without FEX pre-treatment and with one-hour FEX pre-treatment. **(A)** Repeated (2 days) basal challenge to 100 µM HIS in the MucilAir™-pool for IL-6. Data with FEX pre-treatment are expressed in % of vehicle-treated cultures (0.01% DMSO), and HIS data are expressed in % of untreated cultures (the control was vehicle-treated cultures, 6–8 independent experiments, n = 29, HIS n = 51, without FEX pre-treatment n = 21, and with FEX pre-treatment n = 19 cultures, mean + SEM, ordinary one-way ANOVA with Dunnett’s multiple comparisons test *versus* control or HIS, unpaired *t*-test between with and without FEX pre-treatment). Note that there is no significant difference between the vehicle-treated and untreated data. **(B)** Repeated (2 days) basal challenge to 100 µM HIS in MucilAir™-pool for IL-8. Data with FEX pre-treatment are expressed in % of vehicle-treated cultures (0.01% DMSO), and HIS data are expressed in % of untreated cultures (the control was vehicle-treated cultures, 6–8 independent experiments, n = 29, HIS n = 51, without FEX pre-treatment n = 22, and with FEX pre-treatment n = 19 cultures, mean + SEM, ordinary one-way ANOVA with Dunnett’s multiple-comparison test *versus* control or HIS, unpaired *t*-test between with and without FEX pre-treatment). *p < 0.05, **p < 0.001, ***p < 0.001, and ****p < 0.0001.

Without FEX pre-treatment, there was slightly reduced secretion of IL-6 and significantly reduced secretion of IL-8 at day 2 (*p* < 0.0001; Figure 5). Similar anti-inflammatory effects were observed with BIL when used concomitantly with HIS, i.e., a reduction in IL-6 and IL-8 (data not shown). In the side-by-side comparison of FEX in both conditions, one-hour FEX pre-treatment showed significantly higher downregulation of IL-8 (*p* < 0.05) and higher downregulation of IL-6 *versus* without FEX pre-treatment; the anti-inflammatory effect of FEX improved by 22.2% and 39.7%, respectively (Figure 5). Notably, cytokine levels were close to those observed for the controls. For the IL-6 data, the control varied from 52 pg/mL to 2272 pg/mL, and the sample with HIS varied from 452 pg/mL to 4317 pg/mL. For the IL-8 data, the control varied from 8 pg/mL to 64 pg/mL, and the sample with HIS varied from 53 pg/mL to 186 pg/mL. A similar effect was observed from 1 week to 1 h with FEX pre-treatment (data not shown).

**FIGURE 6:**
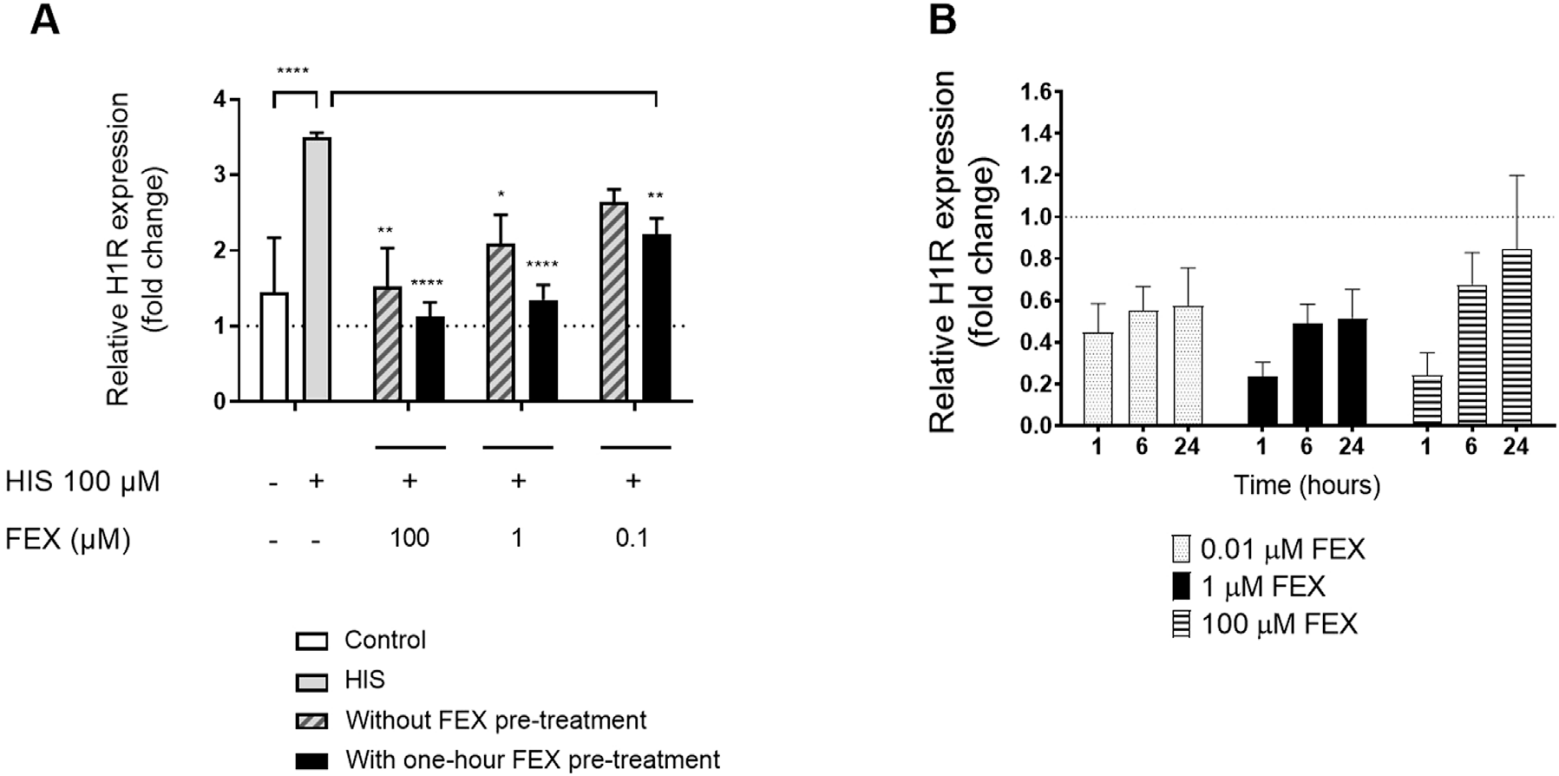
Side-by-side comparison of H1R gene expression without FEX pre-treatment and with one-hour FEX pre-treatment **(A)** and with FEX alone at different concentrations **(B)**. **(A)**. Relative gene expression at 6 h is calculated by normalization of the GAPDH gene and relative to untreated cultures (fold change) (n = 3, mean + SEM, unpaired *t*-test between control and HIS, ordinary one-way ANOVA with Dunnett’s multiple-comparison test *versus* HIS for with and without FEX pre-treatment). **(B)** Relative gene expression is calculated by normalization of the GAPDH gene and relative to untreated cultures (fold change) (n = 3, mean + SEM). *p < 0.05, **p < 0.001, and ****p < 0.0001.

There was reduced H1R gene expression in a dose-dependent manner at 6 h (Figure 6A) in comparison to HIS challenge alone. Similar anti-inflammatory effects were observed when BIL was used concomitantly with HIS, i.e., a reduction in IL-6 and IL-8 (data not shown). There was also higher, dose-dependent downregulation of H1R gene expression with one-hour FEX pre-treatment *versus* without FEX pre-treatment 6 h after the initial HIS challenge (Figure 6A). At the concentration of FEX tested (1 µM), there was an improvement of 35.5% *versus* without FEX pre-treatment, consistent with the improvement observed with other biomarkers. Additionally, there was a trend of downregulation of H1R gene expression when the reconstituted nasal tissue was treated with FEX alone (Figure 6B).

## 4 Discussion

A specific, *in vitro* model of HIS-induced inflammation based on the fully reconstituted human nasal epithelium tissue with relevant biomarkers for HIS-induced inflammation was successfully established to evaluate the effect of antihistamines. The results of this study suggest that IL-8, IL-6, and H1R gene expression levels are promising biomarkers of a HIS-induced inflammation response. HIS at 100 µM induced a rapid upregulation of the H1R mRNA (which normalized at 24 h) and inflammatory biomarkers, demonstrating a relationship between the activation of H1R and the inflammatory response induced by HIS. For the first time using this model with specific biomarkers of activity, the anti-inflammatory benefit of FEX as an inverse agonist was also demonstrated, i.e., able to exhibit dual actions (inhibition of basal activity of H1R and its activation by HIS) and higher activity with additional FEX pre-treatment versus without FEX pre-treatment. Previous studies have used reconstituted nasal tissue as respiratory models for infectious diseases or conditions that may affect the airway (Reus et al., 2014; Boda et al., 2018; Pizzorno et al., 2020); our model is the first to allow exploration of HIS-induced inflammation in reconstituted human nasal mucosa as well as the activity of inverse agonists and the link to clinical benefit.

To assess the effect of FEX as an inverse agonist and its benefit in pre-treatment, we first developed and validated a novel model of HIS-induced inflammation. FEX and BIL were used as positive controls in this newly established model and successfully downregulated the expression of selected biomarkers of inflammation (IL-6 and IL-8) induced by HIS. The results observed with FEX and BIL support the validation of the model and demonstrate the ability of such antihistamines to inhibit the release of pro-inflammatory cytokines induced by HIS in this nasal epithelium model. Additionally, H1R gene expression was proven to be a relevant biomarker of HIS activity in this model; the results demonstrate dose-dependent inhibition of H1R gene expression by FEX. These inflammatory cytokines and H1R gene expression are relevant inflammatory and allergy-related biomarkers that have previously been identified from *in vitro* studies and in clinics (Abdelaziz et al., 1998; Ferreira, 2003; Park et al., 2014; Kitamura et al., 2015). The effect of FEX on inflammatory biomarkers was consistent with previous studies: specifically, inhibition of the eosinophil-induced release of IL-8 from human nasal epithelial cells was significantly attenuated by treatment with FEX, and IL-6 production was significantly reduced by FEX in a nasal fibroblast model (Abdelaziz et al., 1998; Park et al., 2014). Basolateral challenge of the epithelium with HIS induced upregulation of pro-inflammatory cytokines as IL-6 and IL-8; however, other major cytokines involved in the allergic reaction (IL-25, IL-33, and TSLP) were not detectable in the epithelium with or without the addition of HIS. This may suggest an alternative mechanism for the allergic response in the respiratory system, and these cytokines cannot be ruled out at this stage. Notably, the concentration of FEX selected and tested (1 µM) is within the range of the efficacious dose of FEX measured in plasma (approximately 427 ng/mL) (European Medicines Agency, 2021). The results determined using this model may therefore be reflective of the benefits seen with this dose of FEX in clinics.

The presence of FEX in both conditions reduced expression of H1R, IL-6, and IL-8. The results also show that FEX reduces the constitutive receptor activity of H1R in the absence of HIS, confirming the inverse agonist dual mode of action and its benefit when used with pre-treatment. These data are in agreement with a recent article reporting that FEX works as an inverse agonist able to inhibit the basal activity of H1R, based on a HeLa cell line system that express H1R endogenously (Mizuguchi et al., 2020). Furthermore, side-by-side comparison of the conditions showed higher anti-inflammatory activity of FEX (reduction of IL-6 and IL-8 expression) and a trend of higher downregulation of H1R expression with one-hour FEX pre-treatment *versus* without FEX pre-treatment. Additionally, the overall activity of FEX improved in the range of 22% to 40% with pre-treatment. These new findings correlate with the inverse agonist FEX mode of action and the hypothesis stated (Figure 1B), in which HIS-induced H1R activation may be prevented by FEX as it binds the inactive form of the receptor and shifts the equilibrium to the inactive state with higher potency ahead of its activation by HIS.^9^

Currently, in addition to the Allergic Rhinitis and its Impact on Asthma guidelines, allergy society guidelines, health services, and patient advice groups for the management of AR recommend starting treatment before the start of pollen season for better management of AR (ACAAI, 2017; MedlinePlus, 2022; Bousquet et al., 2008; American Academy of Allergy Asthma and Immunology AAAACI, 2021). However, there is limited pre-clinical evidence of the antihistamine mode of action available to support the benefit of this pharmacological approach for protection against and treatment of AR, and it has not yet been shown with FEX. To our knowledge, this is the first pre-clinical evidence of FEX in the context of inverse agonist activity and its link to clinical benefit when used before HIS challenge.

It has previously been shown in a clinical study that pre-treatment with FEX 1.5 h prior to cat allergen challenge significantly mitigated the worsening of rhinitis symptoms associated with cat allergens in humans (Berkowitz et al., 2006). In addition, a study in Japan evaluated the effect of beginning treatment with FEX before the start of cedar pollinosis *versus* early into the season. The study found that starting FEX before the beginning of the pollen season reduced symptoms to a greater extent than beginning treatment with FEX only after the season had already begun.^26^ These clinical findings are supported by the results of this study via specific biomarkers of systemic inflammation previously associated with inflammation in a clinical setting. Given that H1R gene expression level has been linked to symptom severity (Panliang, 1988; Iriyoshi et al., 1996; Mizuguchi et al., 2012; Kitamura et al., 2015; Mizuguchi et al., 2021), the significant inhibition of H1R with pre-treatment suggests that severity of symptoms could be greatly reduced if FEX is taken prior to and during allergen challenge. The results suggest that starting FEX administration prior to any allergen challenge (before H1R activation and the start of symptoms) or when the first symptoms are starting (after H1R activation) could have more of a protective effect and may lessen the intensity of nasal symptoms during allergic episodes.

HIS-induced inflammation in *in vitro* nasal epithelium could be a useful platform to study or screen anti-inflammatory compounds or new modalities. This study has some limitations as it does not consider or examine the other mediators, signaling pathways, or cell types (e.g., immune cells) in an allergic cascade reaction which need to be assessed via *in vitro* studies and in clinics. For example, the basolateral challenge of the epithelium with HIS induced upregulation of IL-8 and IL-6, which are known to activate neutrophils and influence the production of T helper cells and T regulatory cells, respectively (Bickel, 1993; Neveu et al., 2010). In addition, previous studies also show that FEX has a receptor-independent anti-inflammatory effect via inhibition of mast cell and basophil histamine release, as well as inhibition of inflammatory cell activation (Leurs et al., 2002). It should also be considered that cell samples used in the model were not necessarily from allergic patients. Lastly, the model largely represents early allergic inflammation events in the absence of immune cells.

Overall, this study has demonstrated a functional, novel model of HIS-induced inflammation in reconstituted airway tissue and found that IL-8, IL-6, and H1R gene expression levels are promising biomarkers for assessing inflammation and HIS-induced responses in this model. Furthermore, the study provides new insight into the role of FEX as an inverse agonist in this manner and demonstrates the beneficial effect when FEX is taken in anticipation of allergen exposure—when FEX is used before the activation of H1R by HIS or the start of the symptoms. This supports, mechanistically, the preliminary observations from clinics: taking FEX ahead of allergen exposure or at the start of symptoms could have an additional protective effect against HIS activation and subsequent symptoms. This increase in inactive forms of H1R may “shield” the cells downstream in an allergic cascade, helping to lessen symptoms of AR during the pollen season. This is consistent with a previous clinical study using a pollen chamber, which showed that an antihistamine with inverse agonist activity inhibited the basal activity of H1R and showed higher activity when used as prophylactic treatment (Kitamura et al., 2015). Furthermore, these data highlight that human *in vitro* models can predict clinically relevant characteristics of antihistamines and demonstrate their strong translational powers.

## Data Availability

The original contributions presented in the study are included in the article/supplementary material, further inquiries can be directed to the corresponding author/s.
