# Supplementary material for: Establishment of a human nasal epithelium model of histamine-induced inflammation to assess the activity of fexofenadine as an inverse agonist and its link to clinical benefit
Source: Front Pharmacol. 2024 Jun 12;15:1393702. doi: 10.3389/fphar.2024.1393702 (PMC11200123; doi:10.3389/fphar.2024.1393702)
Supplement: Supplementary file 1 [file DataSheet1.docx]

Supplementary Material

Establishment of a human nasal epithelium model of histamine-induced inflammation to assess the activity of fexofenadine as an inverse agonist and its link to clinical benefit

Anne Barbot^1*^, Michele Lheritier-Barrand^1^, Margarita Murrieta-Aguttes^1^, Maud Leonetti^2^, Jimmy Vernaz^3^, Song Huang^3^, Samuel Constant^3^, Bernadett Boda^3^

^1^Sanofi, CHC Scientific Innovation, Neuilly France

^2^Sanofi R&D, Vitry-sur-Seine, France

^3^Epithelix, Plan-les-Ouates, Geneva, Switzerland

*** Correspondence:**Anne Barbot
anne.barbot@sanofi.com

# Supplementary Figures and Tables

## Supplementary Figures


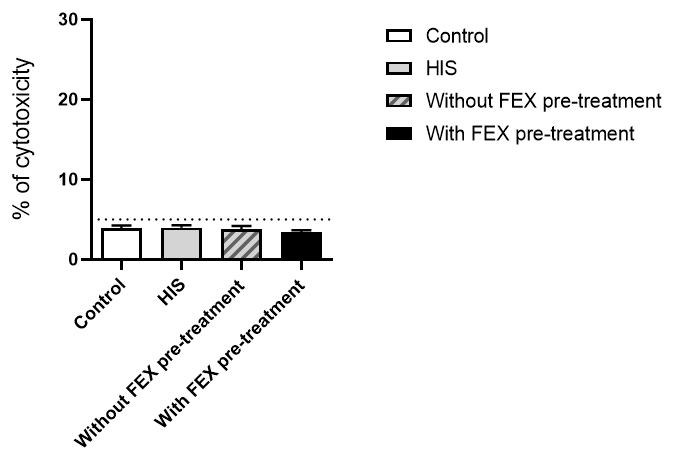


**Supplementary Figure 1.** Lactate dehydrogenase release from plasma membrane of nasal epithelium tissue.

Untreated control shows low daily basal LDH release, <5 %, which is due to a physiological cell turnover in MucilAir™. Below this threshold, the data are considered an absence of cytotoxicity.
